# Disease of Infancy and Childhood

**Published:** 1876-04

**Authors:** 


A Treatise on the Diseases of Infancy and Childhood. By J. Lewis Smith, M.D., Physician to New York Infants' Hospital, Catholic Foundling Asylum, to Protestant Infant Asylum, Clinical Lecturer on Diseases of Children in Bellevue Hospital Medical College. Third edition, enlarged and thoroughly revised, with illustrations. Philadelpbia: Henry C. Lea. 1876.

This work, containing over 700 pages, is again before the profession for popular favor. It contains 100 pages of additional matter, besides an alteration in size of type and illustrations.

Among the diseases now treated for the first time are rotheln and cerebro-spinal fever, epidemics which have occurred in some parts of the country since the former edition of this work. Also an interesting chapter on diphtheria, containing the latest developments in the pathology and treatment of that disease, which has become the terror of all households it invades. This work we consider without a superior, either in this or the oriental world.

